# AAV Vector-Mediated Gene Delivery to Substantia Nigra Dopamine Neurons: Implications for Gene Therapy and Disease Models

**DOI:** 10.3390/genes8020063

**Published:** 2017-02-08

**Authors:** Katrina Albert, Merja H. Voutilainen, Andrii Domanskyi, Mikko Airavaara

**Affiliations:** Institute of Biotechnology, University of Helsinki, Helsinki 00014, Finland; katrina.albert@helsinki.fi (K.A.); merja.h.voutilainen@helsinki.fi (M.H.V.); andrii.domanskyi@helsinki.fi (A.D.)

**Keywords:** adeno-associated virus, alpha-synuclein, Parkinson’s disease, substantia nigra, dopamine, neurotrophic factors, GDNF, striatum, gene therapy, GFP

## Abstract

Gene delivery using adeno-associated virus (AAV) vectors is a widely used method to transduce neurons in the brain, especially due to its safety, efficacy, and long-lasting expression. In addition, by varying AAV serotype, promotor, and titer, it is possible to affect the cell specificity of expression or the expression levels of the protein of interest. Dopamine neurons in the substantia nigra projecting to the striatum, comprising the nigrostriatal pathway, are involved in movement control and degenerate in Parkinson’s disease. AAV-based gene targeting to the projection area of these neurons in the striatum has been studied extensively to induce the production of neurotrophic factors for disease-modifying therapies for Parkinson’s disease. Much less emphasis has been put on AAV-based gene therapy targeting dopamine neurons in substantia nigra. We will review the literature related to targeting striatum and/or substantia nigra dopamine neurons using AAVs in order to express neuroprotective and neurorestorative molecules, as well as produce animal disease models of Parkinson’s disease. We discuss difficulties in targeting substantia nigra dopamine neurons and their vulnerability to stress in general. Therefore, choosing a proper control for experimental work is not trivial. Since the axons along the nigrostriatal tract are the first to degenerate in Parkinson’s disease, the location to deliver the therapy must be carefully considered. We also review studies using AAV-α-synuclein (α-syn) to target substantia nigra dopamine neurons to produce an α-syn overexpression disease model in rats. Though these studies are able to produce mild dopamine system degeneration in the striatum and substantia nigra and some behavioural effects, there are studies pointing to the toxicity of AAV-carrying green fluorescent protein (GFP), which is often used as a control. Therefore, we discuss the potential difficulties in overexpressing proteins in general in the substantia nigra.

## 1. Introduction

Our goal is to review adeno-associated virus (AAV) vector-based gene therapy approaches to overexpress neurotrophic proteins in the striatum or substantia nigra and discuss the use of AAVs as tools to produce Parkinson’s disease models in animals. AAV vector has several characteristics that favour its use for gene therapy in humans. The main advantage is that it is a non-replicating vector that rarely integrates into the host genome, and, indeed, a wild-type (WT) AAV is not known to cause any disease in humans, making it one of the safest viral vectors of choice for gene delivery to the brain [1]. In addition to safety, AAV is able to efficiently transduce various cell types and confer long-term expression in post-mitotic cells in the brain [2]. However, it is possible to insert only a limited size of transgenic DNA, up to 4.7 kb, slightly restricting the applicability [3]. Different combinations of AAV serotypes and promoters can mediate the transduction of different neuron types with varying transgene expression levels in the brain. AAV serotype-2 (AAV2) has been most commonly studied in the rodent brain [4,5,6] and used in clinical trials, though there has been evidence that AAV5 may diffuse better in the brain parenchyma and transduce neurons more efficiently [6]. Additionally, serotypes may be combined together in different ratios to change expression and transduction [7]. With many AAV options available (serotype, promoter, titer, volume), as well as with consideration of how the protein of interest will interact with the target cells, outcomes can vary [3].

Among degenerative brain diseases, Parkinson’s disease has been the focus for gene therapy [3], and as such there are currently seven ongoing clinical trials for Parkinson’s disease using AAVs [8]. In Parkinson’s disease, the nigrostriatal dopamine neurons that project from substantia nigra to the striatum [9,10] degenerate during disease progression [11]. The death of dopamine neurons in the substantia nigra results in the cardinal motor symptoms of the disease such as tremor, rigidity, bradykinesia, and postural instability [12]. A number of factors contribute to the neurodegeneration, including disturbances in mitochondrial respiration [13], environmental risk to certain toxins [14], chronic endoplasmic reticulum stress, and gene mutations [15]. Though the etiology of sporadic Parkinson’s disease (85%–90% of cases [16]) is currently unknown, it seems that the substantia nigra dopamine neurons are particularly vulnerable to stress and protein aggregation [10]. This population of neurons has higher intrinsic levels of metabolic stress and also mitochondrial dysfunction, partially due to the oxidation of dopamine [17]. Additionally, increased intrinsic stress vulnerability of substantia nigra dopamine neurons, as compared to ventral tegmental area dopamine neurons, could be due to their distinct autonomous rhythmic activity at a low frequency with broad action potentials [18].

Regarding the genetic causes of Parkinson’s disease, some of the familial forms are related to mitochondria dysfunction or α-synuclein (α-syn) mutations and duplications or triplications of the α-syn locus [19,20]. Interestingly, the pathological hallmark of both sporadic and genetic forms of Parkinson’s disease are the Lewy bodies, inclusions containing α-syn and many other proteins [21]. Moreover, Lewy pathology is also observed in non-dopaminergic neurons during Parkinson’s disease progression [22].

Results from various studies suggest that the neuroprotective and neurorestorative properties of neurotrophic factors have great potential as a new disease modifying therapy for Parkinson’s disease [23]. A seminal study showed that long-term overexpression of the glial cell line-derived neurotrophic factor (GDNF) protein using recombinant AAV-GDNF driven by the MD (a combination of CMV (cytomegalovirus) enhancer and part of human β-globin) promoter in the rat striatum before a 6-hydroxydopamine (6-OHDA) lesion led to protection of the nigrostriatal system [24]. AAV-GDNF under control of the CMV promoter injected after the 6-OHDA lesion into the rat striatum also showed functional recovery of the nigrostriatal system [25]. Both of these studies showed behavioural recovery on drug-induced rotations and cylinder test assays, two tests often used to measure the severity of a unilateral lesion in rat Parkinson’s disease models. AAV-GDNF has been effective in promoting functional recovery not only in rat models but also in primate models of Parkinson’s disease [26,27] and is currently in a clinical trial coordinated by the NIH Clinical Center [28,29]. Another AAV-based neurotrophic factor therapy for human Parkinson’s disease patients has been neurturin (NRTN), a protein in the same family as GDNF [30]. AAV-NRTN has been shown to be safe as well as efficacious for targeting the substantia nigra in rats and primates, and it was effective in improving the outcomes of a rat 6-OHDA model [31]. However, when AAV2-NRTN was given to the putamen (striatum in rodents) [32] or simultaneously to the putamen and substantia nigra [33] in a double-blind, randomized, controlled trial in parkinsonian patients, it did not show clinically significant benefits over sham surgery. Though interestingly, secondary analysis of a double-blind controlled Phase 2b clinical study of AAV-NRTN identified a more robust response to AAV-NRTN in Parkinson’s disease patients diagnosed within five years prior to treatment, relative to those diagnosed ten years prior or more [34]. In addition, a recent study has identified NRTN variants that may be more effective [35]. Considering gene therapy for Parkinson’s disease patients other than neurotrophic factors, AAV carrying glutamic acid decarboxylase (AAV2-GAD) was administered to the subthalamic nucleus and showed a significant benefit over sham surgery [36]. AAV-AADC (aromatic l-amino decarboxylase, the enzyme that converts levodopa to dopamine) has also shown clinical promise on motor symptoms in particular [37] and is currently in clinical trials [38]. There have also been studies in animals with AAV-TH (tyrosine hydroxylase) or AAV-TH combined with AAV-AADC [39] and with AAV-TH combined with GTP cyclohydrolase I [40] that have shown success. These studies also clearly demonstrated the safety of AAV as a vehicle for protein expression.

The trials described above bring up an important aspect of human gene therapy when targeting dopamine neurons for restoration purposes: whether to target the striatum/putamen or the substantia nigra. This is especially relevant since in Parkinson’s disease the axons along the nigrostriatal tract are degenerating before the dopamine neuron bodies in the substantia nigra [41], and it is not clear how axonopathy impacts the efficacy of functional restoration after striatum/putamen administration. Also, it is likely that the optimal location, striatum/putamen or substantia nigra, as anatomical target for AAV injection is different depending on the therapeutic protein [23].

## 2. Delivering Neurotrophic Factor-Encoding Genes Carried by AAV to the Striatum

Targeting the striatum in gene therapy has been a common approach with neurotrophic factors. Though as with any AAV, there is the issue of specificity and spreading. For example, in a previous clinical trial, the spreading of AAV2-NRTN was limited when injected to the putamen and particularly so when injected to the substantia nigra [42]. However, vectors carrying neurotrophic factor genes that were injected into the striatum can be transported retrogradely to the substantia nigra. For example, in the primate brain, AAV9-carrying green fluorescent protein (GFP) injected to the putamen has been shown to be transported in the anterograde and retrograde directions [43]. This has also been particularly true for AAV2-GDNF [44]. GDNF gene therapy targeting the striatum has restored nigral neurons after 6-OHDA injections in rats, but how AAV retrograde transport to the substantia nigra affects the therapeutic efficacy is unknown [24,45,46,47]. The results of limited efficacy for the AAV-NRTN trial when injected into putamen, together with findings that four to five years after diagnosis of Parkinson’s disease there are few tyrosine hydroxylase (TH) fibres left in the putamen [48], led to clinical trials targeting the substantia nigra [31]. This brings up the question of whether striatally injected AAVs can restore dopamine neurocircuitry only when there are dopamine fibres that can transport neurotrophic factors from the striatum to the substantia nigra. However, in the absence of functional nigrostriatal fibres, GDNF expressed in striatal medium spiny projection neurons can be anterogradely transported to the substantia nigra pars reticulata and may thus signal locally in the substantia nigra on the nearby substantia nigra pars compacta dopamine neurons [49]. Moreover, there is a large body of experimental evidence indicating that striatal AAV delivery may be better than delivery into the substantia nigra. A neuroprotective study in mice with striatal AAV-GDNF injection against MPTP (1-methyl-4-phenyl-1,2,3,6-tetrahydropyridine)-induced experimental parkinsonism showed preserved synaptic plasticity and protected against the loss of dopamine; however its transport was not measured here [50]. AAV-GDNF in the 6-OHDA model of Parkinson’s disease has shown efficacy in both neuroprotective and neurorestorative studies in rats. When AAV-GDNF was injected before the 6-OHDA to striatum, in both cases it was able to protect substantia nigra dopamine neurons [24,51]; perhaps unsurprisingly since the tract likely remained intact. Only the striatal delivery led to the functional recovery of the rats, whereas substantia nigra delivery in the same study resulted in substantia nigra dopamine neurons remaining protected but no recovery of behaviour [24]. More importantly, when AAV-GDNF was infused to the striatum after the 6-OHDA, this also led to behavioural recovery as well as the restoration of dopamine neurons due to retrograde transport to the substantia nigra [25]. AAV-GDNF can also be expressed in monkey substantia nigra via axonal transport when delivered to the putamen [27]. Similar to AAV-GDNF, AAV2-NRTN delivered to rat striatum before 6-OHDA showed neuroprotection in the substantia nigra dopamine neurons [51]. AAV2-NRTN striatal delivery in monkeys led to its expression in both the striatum and substantia nigra, as well as increased levels of TH in the nigrostriatal system [52,53]; though again this is not surprising but does demonstrate the ability of AAV2 carrying NRTN to also transport to the substantia nigra after striatal injection. As in rats, AAV2-NRTN injected to both the striatum and substantia nigra has been shown to be neurorestorative in the monkey MPTP model [54], indicating its ability to be transported after the toxin damage and result in neuronal recovery. There is also evidence of recovery with another neurotrophic factor: CDNF (cerebral dopamine neurotrophic factor) carried by AAV2. When AAV2-CDNF was injected before [55] and after [56] the 6-OHDA to the striatum, it showed the recovery of striatal fibres as well as nigral neurons in addition to behavioural recovery, and CDNF protein was present in both structures.

In general, while the dopamine nerve terminals are destroyed in the striatum/putamen after toxin administration, transport of neurotrophic molecules to the substantia nigra may still be possible retrogradely, as described above, or anterogradely via medium spiny projection neurons, thus demonstrating that striatal injection of AAV carrying neurotrophic factors is a viable therapy for Parkinson’s disease. Although, the anterograde transport of GDNF to the substantia nigra may not be as effective on functional restoration as compared with retrograde transport, it may be beneficial not to overexpress proteins in stressed substantia nigra dopamine neurons in patients.

## 3. AAV to Target Substantia Nigra

Taking into consideration the special characteristics of substantia nigra dopaminergic cells (see above), targeting these neurons with AAV vectors can be challenging. For example, if a promoter driving a gene of interest is giving exceptionally high expression levels, this can naturally cause excessive protein accumulation and lead to further stress on the cells [23]. Safe transduction of the dopamine neurons in the substantia nigra is an important aspect in therapeutic approaches, and there have been several studies testing AAV preparations to target substantia nigra dopamine neurons. It has been demonstrated that injection of AAV-GFP to the substantia nigra of rats showed long lasting expression in both the nigral areas (up to 25 months) and striatum (up to 18 months) [57]. Similar results have been obtained in mice, with various vector serotypes able to label the whole substantia nigra, ventral tegmental area, as well as the striatum [58]. In regards to AAV-GFP and dopamine neurons in the substantia nigra, studies in rats have shown that several different vector preparations carrying GFP are able to transduce TH-positive neurons effectively in the substantia nigra as well as in fibres of the striatum [59,60]. Transduction efficiency of AAVs in substantia nigra neurons has also been tested successfully in monkeys, a relevant model when considering the clinical implication of viruses since the size differences between the rodent, primate, and human brain need to be taken into account, particularly concerning spreading. As such, studies have shown that injecting AAV-GFP to the substantia nigra of monkeys results in efficient transduction of the neuronal [61] and dopaminergic cells [62] of the substantia nigra. It is clear that choosing the virus serotype is dependent on the species and the areas or neurons that need to be transduced; therefore, selecting the correct preparation for one′s experiment will depend on these factors. It is also evident that it is possible to transduce TH neurons in the substantia nigra with AAVs successfully.

In relation to neurotrophic factors, though they are usually given to the striatum, a study compared striatal and nigral delivery in rats where the AAV-NRTN was given simultaneously with the 6-OHDA lesion. This was performed with an idea that enhancing the survival of the substantia nigra neurons by nigral AAV-NRTN injection may induce greater protection of the connecting striatal fibres. Indeed, the results showed that targeting both the striatum and substantia nigra was the most effective strategy [63]. However, in the case of therapeutic proteins, the overall detrimental effects of overexpression of transgene or RNA can be ‘hidden’ by the positive effect of the therapy itself. Specifically, using only a non-AAV control may not show the damaging effects. Moreover, caution should be exercised when trying to overexpress any proteins in dopamine neurons, as this can induce stress response pathways affecting their functions and survival, as in [64] and [65]. As a way to overcome the detrimental effect of constitutive protein overexpression or to regulate signalling of neurotrophic factors that are known to be very potent, regulated vectors have been developed. A destabilizing domain that can be regulated with trimethoprim has been effectively used with AAVs carrying GDNF [66]. A single AAV vector for tetracycline-regulated expression with rtTA (reverse tetracycline transactivator) has also been used for GDNF transgene expression in Parkinson’s disease models [67]. Mifepristone-controlled vectors have also been shown to restore motor function in the 6-OHDA rat model of Parkinson’s disease [47,68]. The mifepristone-regulated vectors are based on binding to a human progesterone receptor, fused to a human p65 transactivation domain and a yeast Gal4 DNA-binding domain. As endogenous neurotrophic factors are biologically active at very low doses, these regulated approaches could be used for neurotrophic factor therapies and may be safer options for targeting substantia nigra dopamine neurons. However, similar to the regulated vectors that express any other foreign protein, there is caution to be considered since there are indications of astrocyte expression–mediated toxicity and a widespread inflammatory response caused by non-self transgenic protein overexpression [69].

## 4. AAV-α-syn Animal Models of Parkinson’s Disease

Transducing substantia nigra dopamine neurons with genes related to human Parkinson’s disease has been explored to provide valuable animal models of the disease. AAV carrying cDNA of human WT α-syn injected to the substantia nigra induces slowly progressing neurodegeneration and the presence of α-syn inclusions [70,71]. These models have been used in mice [72,73,74] and rats. A study using both human WT or mutant A53T α-syn carried in recombinant AAV-CBA (chicken β-actin) injected into the substantia nigra of rats showed a loss of striatal TH fibre density and dopamine and degeneration of TH-positive cells in the substantia nigra. Furthermore, in animals with a significant loss of TH in the nigral area, severe behavioural deficits developed at 24–27 weeks post-injection, as compared with GFP controls [75]. Studies using WT human α-syn with AAV6 [76], AAV5 [77], and AAV9 [78] injected to the nigra have shown TH loss in the nigrostriatal system and motor deficits. In addition, recombinant AAV human WT α-syn injection to rats has resulted in the phosphorylation of endogenous α-syn in the substantia nigra area [79]. However, in [80], the authors observed that α-syn phosphorylated at Ser129 is not necessary for pathology in the dopamine system and deficits in motor behaviour and therefore, WT human α-syn is enough to cause dysfunction. For the mutant forms, AAV5-S129D and S129A [80], AAV2-A53T [81], and AAV6-A30P [82] have been tested in rats and compared to the WT form. A large study compared the model in mice, rats, and monkeys, wherein AAV9 human mutant A53T α-syn was injected into the substantia nigra, and found that there was degeneration in the nigrostriatal areas in all species and motor deficits in rats, but interestingly aging did not exacerbate the loss or symptoms [83]. A long-term study was performed with a variety of pathology and behavioural measures, in which the authors used AAV7 carrying human WT or mutant (A53T) α-syn and injected it into the substantia nigra [84]. The results showed that this mutant form was able to induce behavioural deficits on rotarod and cylinder tests, induce dopamine cell death in the substantia nigra, decrease dopamine release in the striatum, and also form insoluble aggregates [84]. A recent study in rats using human WT α-syn with four and eight week time points did not show TH loss in the striatum at 4 weeks, but did show a mild loss of TH-positive neurons in the substantia nigra and decreased dopamine content at eight weeks after vector injections [85]. While most of these studies do show some loss of TH and robust α-syn expression, which are important in modelling the disease, in some cases, AAV carrying GFP or other control results were not shown as a comparison. Additionally, many of these studies use enhancers for the transgene expression levels. It should also be noted that it is a mutant form of human α-syn in certain studies, not the WT, which demonstrates TH loss and motor deficits in the animals. In Table 1 we have summarized these studies, in which a GFP control was most often used as a comparison to the AAV-α-syn.

In general, though some of the previous studies were able to recapitulate the disease, there is still large variability between and within studies, including our own findings [65]. Additionally, without a control for a comparison of dopamine neuron loss in the substantia nigra, it is difficult to conclude that it is specifically the α-syn overexpression causing the damage. By optimizing the volumes, particle number and injection needle parameters to be similar as described in, for example, [75], there is no reason why the number of successfully lesioned rats with AAV-α-syn could not be increased. However, proper controls need to be used for comparison and it is clear that caution should be applied when setting up the model.

## 5. Challenges in Control AAV Vectors for Targeting Substantia Nigra Dopamine Neurons

**Figure 1:**
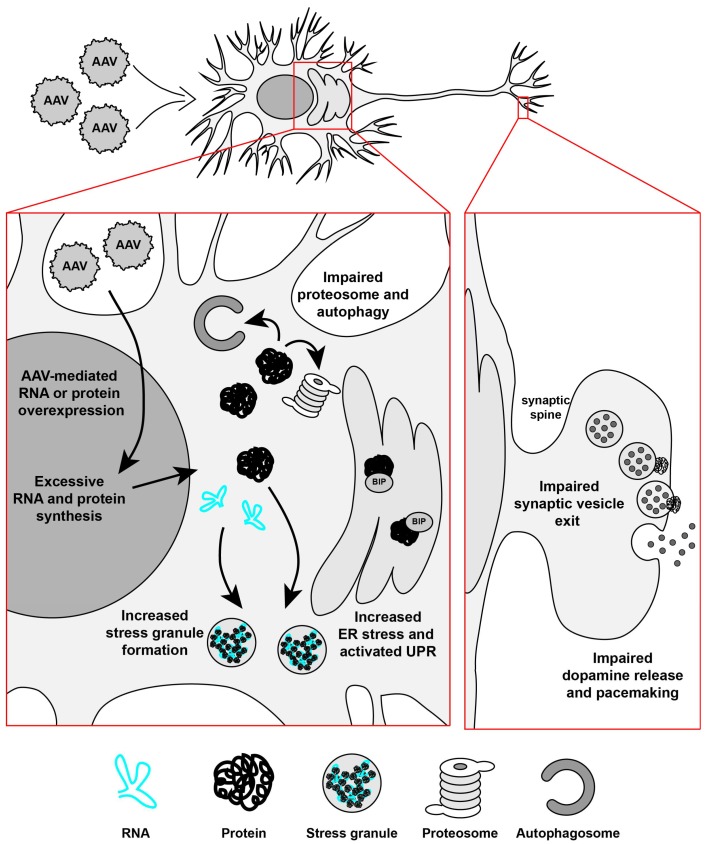
Schematic diagram of protein overexpression via AAV in the substantia nigra dopamine neurons. When an AAV carrying protein or RNA is expressed in the brain, particularly in the substantia nigra where dopamine neurons are vulnerable to stress, the consequences of excessive overexpression may result in a number of events detrimental to cell survival. These include the increased formation of stress granules, increased levels of endoplasmic reticulum (ER) stress, activation of the unfolded protein response (UPR), and impairment of the proteasome function and autophagy. This could further result in impairments in vesicle fusion at the synapse and difficulties in the dopamine release and pacemaking functions of the neuron. This would culminate in reduced neuronal activity and may result in the death of the neuron. (BIP: Binding immunoglobulin protein).

To study the AAV-α-syn-mediated mechanisms, it is important to use appropriate controls to ensure that the degeneration is not due to protein or RNA overexpression in general. It is also valuable to study whether the control proteins, RNA, or empty vectors are toxic to the dopamine neurons. As mentioned, substantia nigra dopamine neurons are particularly vulnerable to stress and therefore can be easily damaged. Although AAV vectors produce a minimal immune response in the brain [86], the extent of the cellular antiviral defence response in substantia nigra dopamine neurons after AAV vector injections is not known and not well studied. Innate immune responses triggered by pattern recognition receptors in relation to AAV capsids or by DNA are not known; for example, there have been no studies conducted on the relationship of Toll-like receptors and AAV vectors [87]. However, an important study was performed in which the authors observed that AAV9 mediated the overexpression of GFP, a protein from jellyfish, which resulted in an astrocyte-mediated immune response when injected to the nonhuman primate brain, as opposed to AAV carrying a non-foreign protein, which did not end in a toxic response [69]. Additionally, while increasing the titer of an AAV will naturally increase the amount of positive cells and protein expressed, it can also lead to neurodegeneration. In a study in which AAV8 carrying GFP was injected into the substantia nigra of rats, the high dose used caused a 41% loss of dopamine neurons compared to the non-injected side, however the lower dose showed no neurotoxicity [88]. When a chimeric AAV1/2-GFP was used at the same titer as AAV1/2 carrying A53T α-syn, it also caused neuron death in the substantia nigra of rats, but when the titer was lowered to 1:3 or 1:10, no toxicity was observed with GFP [81]. A recent study compared the titers of AAV-α-syn and AAV-GFP and demonstrated that, at a titer of 2 x 10^14^ gc/mL and higher, GFP resulted in approximately a 62% loss of TH positive cells in the substantia nigra [64]. These studies clearly demonstrate that overexpression-induced dopamine neurodegeneration is not specific to α-syn and careful testing and caution needs to be taken with regard to how much virus is used in the control condition, particularly with neurodegeneration studies. And indeed, it has been previously reported that AAV5-eGFP can cause a significant lesion to the dopamine system as the corresponding AAV5-α-syn vector [89]. In addition, immune responses to non-self proteins need to be taken into consideration. We have found that not only the overexpression of GFP via AAV but also the overexpression of RNA that did not translate to any protein, led to degeneration of dopamine neurons in Parkinson’s disease models [65]. Neuronal loss after AAV-mediated transduction is most likely caused by the increased sensitivity of dopamine substantia nigra neurons to stress, due to their properties [10], and the results clearly suggest that it is not only protein overexpression that can be detrimental but also RNA overexpression. Indeed, excessive RNA and/or protein synthesis after AAV-mediated transduction of nigral dopamine neurons can impair proteosome and autophagy pathways, promote formation of stress granules, activate endoplasmic reticulum, and unfolded protein response pathways, collectively leading to synaptic dysfunctions and impaired neuronal activity (Figure 1). The results described above suggest that such overloading of substantia nigra dopamine neurons with either RNA or protein can be caused by the overexpression of WT, mutant α-syn, GFP, untranslated RNA, or perhaps by any non-specific sequence. Therefore, intranigral injections of AAV vectors for dopamine neuron transduction may not be the best method in neurodegeneration studies.

As far as recommending a control for neurodegeneration research, the optimal control for α-syn should be considered. Using similar titers or particle numbers of α-syn and control viral vectors would be essential to evaluate α-syn specificity on causing degeneration of dopamine neurons. GFP and similar proteins are often used as controls, but, as they themselves can cause neurodegeneration and immune responses, are they really the best controls in all cases? We would also consider using mammalian proteins instead of commonly used fluorescent proteins derived from marine organisms. It would also be worthwhile considering a protein that forms oligomers that are not known to cause neuronal pathology. Endogenous α-syn likely is in soluble oligomeric form [90], and its aggregation and fibril forms may be important in disease pathology due to their toxicity [91]. Thus, it would be important to use proteins that produce non-toxic oligomers to compare the in vivo effects in the substantia nigra of animals. Examples of these proteins are mostly cytoskeletal components such as actin or collagen.

## 6. Conclusions

AAV as a vehicle to deliver both therapeutic proteins and potentially toxic or pathogenic proteins has been widely used and high protein expression is found in the brain even after long periods of time, making it a powerful tool in therapy and disease models. Consideration of whether to inject AAV into the striatum or the substantia nigra due to the intrinsic properties of the substantia nigra dopamine neurons is an important aspect in neurotrophic therapy. There is also evidence of unspecific immune responses or cell death due to protein overexpression in the substantia nigra in general, and therefore using foreign proteins in neurodegeneration studies should be considered closely. After reviewing the literature on previously published AAV-α-syn studies and performing a series of experiments, it is clear that, while it is possible to achieve mild degeneration in this model, caution needs to be taken in selecting appropriate control vectors when targeting the substantia nigra in order to avoid proteinopathy or RNA toxicity [65].

## Figures and Tables

**Table 1:** Summary of articles with human adeno-associated virus (AAV)-α-synuclein unilaterally injected to the substantia nigra of animals where outcomes are compared to AAV-green fluorescent protein (GFP). Animal and strain are listed, along with serotype, promoter, and if any enhancers are used. The total duration of each experiment is listed, though earlier time points may have also been measured. Striatum tyrosine hydroxylase (TH) fibre density is defined as the TH optical density as a percentage of the control side, TH+ cell loss refers to either total cells or a percent of the control side, striatum dopamine content is measured by HPLC, and behaviour refers generally to any behavioural assays that were performed in the experiments.

Laboratory Animal	Insert	Serotype/Promoter	Duration	STR TH Fibre Density	SN TH+ Cells	STR Dopamine	Behaviour	Ref.
C57BL/6 mice	WTA53T	AAV7/CMV (w/WPRE)	8 weeks	N/A	↓	N/A	WT: motor deficits	[72]
C57BL/6 mice	WT	rAAV/CMV (w/WPRE and IRES)	24 weeks	N/A	↓	N/A	N/A	[73]
Sprague Dawley rats	WT	rAAV/CBA (w/CMV elements)	27 weeks	↓	↓	↓	No motor deficits *	[75]
Sprague Dawley rats	WT	AAV6/CBA (w/and w/out WPRE)	16 weeks	↓	↓	↓	Motor deficits	[76]
Sprague Dawley rats	WT	AAV5/CBA/CMV	8 weeks	N/A	↓	N/A	Some motor and non-motor deficits	[77]
Fisher 344 rats	WT	AAV9/CBA	12 weeks	N/A	↓	N/A	Motor deficits	[78]
Sprague Dawley rats	WT	rAAV/CMV	13 weeks	N/A	↓	↔	No motor deficits	[79]
Sprague Dawley rats	WTS129AS129D	AAV5/CMV/CBA	15 weeks	↓	↓	N/A	Some motor deficit	[80]
Sprague Dawley rats	A53T	AAV1/2/CBA/CMV (w/WPRE and bGH-polyA)	6 weeks	↓	↓	↓	Some motor deficit	[81]
Sprague Dawley rats	WTA30P	AAV6/CMV (w/WPRE)	16 weeks	↓•	↓•	↓•	Some motor deficit	[82]
Wistar rats	WTA53T	AAV7/CMV/synapsin	29 days	N/A	A53T: ↓	A53T: ↓	A53T: Some motor deficit	[84]
Sprague Dawley rats	WT	AAV5/human U6	8 weeks	N/A	↓	↓	No motor deficit	[85]

N/A refers to the fact that an outcome measure was not included in the article; ↓ A decrease in the parameter was shown; ↔ No change in the parameter was shown; * With TH enzyme blocker on stepping test; • Not compared to GFP; STR: striatum; SN: substantia nigra; TH: tyrosine hydroxylase; CBA: chicken β-actin; CMV: cytomegalovirus; WT: wild-type; WPRE: woodchuck hepatitis virus posttranscriptional regulatory element; IRES: internal ribosome entry site; bGH-polyA: bovine growth hormone polyadenylation sequence.

## Abbreviations

The following abbreviations are used in this manuscript:

6-OHDA	6-hydroxydopamine
α-syn	alpha-synuclein
AADC	aromatic l-amino decarboxylase
AAV	adeno-associated virus
BIP	binding immunoglobulin protein
bGH-polyA	bovine growth hormone polyadenylation sequence
CBA	chicken β-actin
cDNA	complementary deoxyribonucleic acid
CDNF	cerebral dopamine neurotrophic factor
CMV	cytomegalovirus
DIO	double-floxed inverse ORF
EF1a	elongation factor 1 alpha
GAD	glutamic acid decarboxylase
GFP	green fluorescent protein
GDNF	glial cell line-derived neurotrophic factor
HPLC	high performance liquid chromatography
IRES	internal ribosome entry site
iRFP	infrared fluorescent protein
MPTP	1-methyl-4-phenyl-1,2,3,6-tetrahydropyridine
NRTN	neurturin
NSE	neuron-specific enolaseRNA: ribonucleic acid
rtTA	reverse tetracycline transactivator
SEM	standard error of the mean
SPECT/CT	single-photon emission computed tomography/computed tomography
TH	tyrosine hydroxylase
WPRE	woodchuck hepatitis virus posttranscriptional regulatory element
WT	wild-type
